# Electrophysiological Prints of Grit

**DOI:** 10.3389/fpsyg.2021.730172

**Published:** 2021-10-14

**Authors:** Nuria V. Aguerre, Carlos J. Gómez-Ariza, Antonio J. Ibáñez-Molina, M. Teresa Bajo

**Affiliations:** ^1^Department of Experimental Psychology—Mind, Brain and Behavior Research Center (CIMCYC), University of Granada, Granada, Spain; ^2^Department of Psychology, University of Jaén, Jaén, Spain

**Keywords:** grit, EEG, theta/beta ratio (TBR), entropy, fractal dimension, impulsiveness

## Abstract

While scientific interest in understanding the grit trait has grown exponentially in recent years, one important gap in the grit literature relates to its biological and neural substrate. In the present study, we adopted a hypotheses-driven approach in a large sample of young adults (*N* = 120) with diverse educational backgrounds and work experiences in order to investigate the electrophysiological correlates of grit both during rest and while performing a learning task. Additionally, we selected a measure of impulsiveness to better understand the neural similarities and differences between grit and related self-control constructs. Based on previous work that implicated the prefrontal cortex in grit, we hypothesized that high grit participants would have lower frontal theta/beta ratio (a broadly used index that reflects prefrontally-mediated top–down processes, which might indicate better control over subcortical information). Furthermore, we expected the perseverance of effort facet of grit to be linked to higher complexity during task engagement because previous research has shown complexity indexes (entropy and fractal dimension) to be linked to effort while performing cognitive tasks. Our results revealed that although there were no differences at rest as a function of grit, the participants with high grit and high consistency of interest scores exhibited lower frontal theta/beta ratios during the learning task. This pattern suggests that individual differences in grit might be more evident when top-down control processes are at work. Furthermore, there was a positive association between perseverance of effort and entropy at task, which might indicate more effort and engagement in the task. Finally, no association was found between the neural indexes (frontal theta/beta ratio, entropy, or fractal dimension) and impulsiveness, neither impulsiveness mediated between grit and brain measures. Finally, when controlling for impulsiveness and demographic variables (gender, age, education, and work experience) the effects at the facet level remained statistically significant. While there is still a long way to fully understand the neural mechanisms of grit, the present work constitutes a step toward unveiling the electrophysiological prints of grit.

## Introduction

In a world in which new information emerges every second, sticking with one dream can be challenging. Indeed, not everybody chooses to strive toward a long-term goal and even fewer people maintain their motivation until they have achieved it. Grit is the personality trait that defines those people that do tend to pursue long-term goals with enduring passion and perseverance (Duckworth and Gross, 2014). This newly explored trait has attracted the attention of researchers from different fields (i.e., positive psychology, motivation, and education), given that it has been shown to be able to predict success in various domains and contexts, such as academic (i.e., Duckworth et al., 2007; Clark and Clark, 2019), work achievement (i.e., Mueller et al., 2017), and personal life (i.e., Eskreis-Winkler et al., 2014). More importantly, grit has been shown to be related to different aspects of well-being (i.e., general well-being: Duckworth et al., 2007; Kindt et al., 2009; Kannangara et al., 2018; Jiang et al., 2020; life satisfaction: Li et al., 2018a; lower depression: Musumari et al., 2018; Datu et al., 2019; reduction of risk of suicidal ideation: White et al., 2017; Kaniuka et al., 2020). While interest in grit has grown exponentially over the past few years, the neural processes underlying this trait still remain largely understudied.

Grit is conceptualized as comprising two factors (Duckworth and Quinn, 2009): *perseverance of effort* and *consistency of interest*. The first factor, *perseverance of effort*, highlights the long-term stamina or the effort maintained toward one's superordinate goal, whereas the *consistency of interest* factor taps into the passion for one's goal and the ability to stay committed to interests related to it. Hence, grit is related to both self-control and motivation (Nemmi et al., 2016). The effortful regulation of attention, emotion, and behavior would allow self-controlled individuals to overcome temptations in comparison to their impulsive counterparts (Duckworth, 2011). This ability would help these individuals to achieve long-term goals as well. Interestingly, evidence indicates that self-control and grit correlate moderately (*r* = 0.63; see Duckworth et al., 2007), which suggests that there must be something else besides self-control in the consecution of long-term goals (Duckworth and Gross, 2014; Eskreis-Winkler et al., 2014; Li et al., 2018b; Tedesqui and Young, 2018). On the other hand, motivation is thought to contribute to how people behave, think, and feel. Individual differences in motivation reflect the degree of endurance in people's needs, desires and values (Borghans et al., 2008) and, therefore, a certain pattern of motivation would be behind grit. In fact, there is evidence that grit is positively linked to orientation toward engagement and inversely associated with pursuing pleasure in Western samples (Von Culin et al., 2014; see also: Muenks et al., 2018). In accordance with this conceptualization, the few studies that have already examined the neural basis of grit converge in showing that grit is mainly associated with the function and structure of the prefrontal cortex (PFC) and striatum, which are the key regions for executive control (self-control) and reward (motivation) processes (Myers et al., 2016; Nemmi et al., 2016; Wang et al., 2017, 2018).

For example, Nemmi et al. (2016) examined brain structure as a function of grit in 27 children and found that individual differences in the trait were associated with differences in the volume of the nucleus accumbens, which has been related to reward-seeking (Tobler et al., 2014). In a resting-state functional magnetic resonance imaging (fMRI) study with 20 children, Myers et al. (2016) found grit to be associated with ventral striatal and bilateral prefrontal networks. The ventral striatum was specifically connected to medial prefrontal and rostral anterior cingulate cortices. Importantly, all these regions are thought to be crucial for cognitive-behavioral control, perseverance, and emotional regulation. More recently, Wang et al. (2017) tested resting-state fMRI in 217 healthy adolescents and found a negative relationship between grit and the regional fractional amplitude of low-frequency fluctuations in the right dorsomedial PFC, which is thought to be involved in self-regulation. Furthermore, this association played a mediating role in the link between grit and academic performance. In a related structural MRI study—also with adolescents—, Wang et al. (2018) found greater volume in the right putamen and smaller volume in the left dorsolateral PFC, both regions involved in action planning, motivation, and self-regulation in gritty participants.

Some other attempts have been made to examine the neural basis of grit by using electroencephalography (EEG). In this regard, Kalia et al. (2018) recorded event-related potentials while participants (undergraduate students) performed the attentional network task (ANT; Fan et al., 2002). Kalia et al. (2018) found that people with higher scores in the *perseverance of effo*rt facet of grit were linked to reduced electrophysiological responses (N1) to an alerting cue relative to people with lower scores. According to the authors, this attenuated alerting effect for grittier individuals might be a sign of their more efficient sustained attention due to their stronger intrinsic motivation to perform well. Thus, alerting cues were less effective as a warning signal, since they were already more attentive to the task. More recently, Matthews et al. (2019) included a measure of grit in a study examining the role of worry and resilience in the performance of 68 undergraduate students in a Unmanned Aerial simulation System including different stress-inducing conditions and physiological measures. Although no EEG results were reported regarding the control condition, in the high stress condition there was an association between (high) grit and (lower) gamma activity that the authors interpreted as indicating that grittier individual might also show better stress coping abilities.

While these results are compelling, a number of factors limit the conclusions that can be drawn from them. First, some of the studies employed very small sample sizes. Second, MRI studies primarily focused on the brain state at rest, although it is also possible that differences occur when gritty people engage in a task due to differential information processing. Third, these studies focused only on grit-related traits, leaving out other self-control traits that could provide information regarding the neural similarities and differences between these constructs. Fourth, all studies focused on children, adolescents or grad students with similar life backgrounds, thus limiting the generalizability of their findings. Finally, most studies have used a single brain dimension, although some authors have pointed out that it is necessary to approach the topic using different brain measures (van Zyl et al., 2021). Hence, more studies that use more heterogeneous and larger samples and that employ distinct brain measures in different conditions are required in order to better understand the grit trait.

In addition, it is relevant to examine how different self-control constructs relate to grit by tapping into their commonalities and differences at the neural level. This point is of particular importance because one major concern about the grit construct has been its dissociation from other concepts related to self-control (Muenks et al., 2017; Schmidt et al., 2018; Vazsonyi et al., 2019; Werner et al., 2019). A key self-control related concept that is thought to be closely related to grit (specifically to its *consistency of interest* facet) is impulsiveness (Schmidt et al., 2018). Impulsiveness is defined as the tendency to perform swift actions without conscious judgment (Patton et al., 1995) and provides an interesting scenario to study the relation between grit and other self-control measures. Impulsiveness is considered to be the opposite of self-control (Duckworth, 2011), but it does not include items related to sensation seeking (Stanford et al., 2009) that are often included in self-control scales and that have been demonstrated to have a distinct nature from impulsiveness or grit (Duckworth and Kern, 2011). Although impulsiveness is conceptually related to the absence of grit, grit is theoretically thought as more complex than just a low impulsiveness pattern (Duckworth and Gross, 2014). In fact, it has been shown that the two constructs are negatively correlated (Grif et al., 2016). However, this relationship is not very strong and the extent to which grit differentiates from impulsiveness is still unknown. Importantly, on some occasions impulsiveness has been shown to predict academic performance beyond grit (Rennicks, 2018). Hence, in the present study we considered impulsiveness when examining the neural underpinnings of grit in order to deal with potential confounding effects.

Furthermore, as stated, it is also convenient to employ heterogeneous samples and take into account the demographic background of the participants when examining the neural bases of grit. This point is of importance because one concern about the existing literature on the neural substrates of grit is the similar and homogeneous samples that the few studies on the topic included, which limits the generalizability of their findings (van Zyl et al., 2021). For this reason, we selected participants of different educational and work background, two variables that have been closely (positively) related to grit (Duckworth et al., 2007; Mueller et al., 2017), and considered these variables when examining the neural underpinnings of grit.

Finally, to gain further understanding of the neural processes underlying grit, a hypotheses-driven approach should be adopted because it allows researchers to conceptually replicate previous findings. In this vein, electroencephalography (EEG) constitutes an adequate technique that provides high temporal resolution and distinct indexes of brain activity, allowing for the formulation of specific hypotheses. Hence, because grit has a strong self-regulation component and because its expression has been linked to activity in the PFC, we focused on a widely used executive control index: the frontal theta/beta ratio (TBR) (Putman et al., 2010, 2014; Angelidis et al., 2016; Syed Nasser et al., 2019). TBR is thought to reflect prefrontally-mediated attentional control and has broadly been used as a biomarker for impulsiveness-related disorders, such as the attention-deficit hyperactivity disorder (Barry et al., 2003; Snyder and Hall, 2006; Lansbergen et al., 2007; Arns et al., 2013; Zhang et al., 2017). High frontal TBR is usually interpreted as failure in exerting top-down control over the automatic processing of subcortical information. Based on previous work that implicated PFC and striatum structures in grit, we hypothesized that high grit participants would have lower frontal TBR, which might reflect better control (top–down processes) over subcortical information (reward information of the striatum). In addition, we aimed to explore whether (1) impulsiveness mediated the possible effect between TBR and grit, and (2) it had a particular TBR pattern dissociable from grit. In the same vein, we also wanted to explore whether the demographic variables of our heterogeneous sample (gender, age, education and work experience) could partially explain the possible association between TBR and grit.

Complementarily, we included a complexity-based approach to the analysis of EEG recordings by tapping into entropy (SampEn) and fractal dimension (HDF) brain indexes. These indexes, based on non-linear assumptions from system theories, are increasingly being recognized as valuable tools for capturing complex brain signals (Costa et al., 2002, 2005; Ouyang et al., 2010). While extreme patterns of complexity at rest can be indicative of pathology (Ibáñez-Molina et al., 2018), complexity indexes have been linked to effort while performing cognitive tasks (Müller et al., 2003; Stam, 2005; Sohn et al., 2010). Given the relationship between *perseverance of effort* and task values, self-efficacy, and general effort (Muenks et al., 2017; Zamarro et al., 2020), it is plausible to hypothesize that high grit participants (high *perseverance of effort* participants in particular) might show higher brain complexity levels during task performance as an indicator of task engagement (Müller et al., 2003; Stam, 2005; Sohn et al., 2010). Again, as with TBR, we explored whether impulsiveness and demographic variables affect this relationship between *perseverance of effort* and complexity indexes.

In sum, in the present study, we adopted a hypotheses-driven approach on a large sample of young adults with diverse educational backgrounds and work experiences in order to investigate the electrophysiological prints of grit. Participants completed the Grit Scale (Duckworth et al., 2007) and underwent EEG recordings at rest and while performing a learning task. Additionally, we selected a measure of impulsiveness to better understand the neural similarities and differences between grit and related self-control constructs. As mentioned, we hypothesized that high grit participants would exhibit lower frontal TBRs (both at rest and while performing a learning task), which might reflect more efficient top-down control over reward processes in comparison to their low grit counterparts. In addition, we expected that participants characterized by high levels of effort on the Grit Scale would also be characterized by greater complexity as an indicator of task engagement (Müller et al., 2003; Stam, 2005; Sohn et al., 2010). Finally, we explored whether impulsiveness and some demographic variables were modulating the effects between grit and the brain.

## Method

### Participants

A total of 120 people (*M*_age_ = 23.11, SD_age_ = 4.19, Range = 18–33, 69% female) completed the study in exchange for course credits (0.1 credit/40 min) or monetary reward (7 €/1 h). Participants differed in educational levels and job backgrounds. In terms of education, 17 participants had only attended secondary school, 58 were enrolled in university courses (toward a variety of degrees), and 45 had already completed a university degree. Of these graduates, 22 were enrolled in master's courses during the time of their participation in the study. With respect to work experience, 54 participants reported that they did not have any professional experience in any field, whereas 66 participants noted that they did have professional experience (i.e., as waiters, researchers, dancers, doctors, etc.). All participants included in the experiment informed in a written health questionnaire to be free from any health issue, neurological problem, drug consumption or cognitive dysfunction diagnosis. The sample was a part of a larger study that focused on individual differences and other non-overlapping findings resulting from that study have already been reported (Aguerre et al., 2020). Participants provided their written informed consent in order to participate in the study, following the Helsinki Declaration guidelines (World Medical Association, 2013), and approval was obtained from the Ethics Committee of the University of Granada.

### Materials and Procedure

Participants were tested individually in two sessions that lasted 90 and 120 min, respectively. In the first session, they were administered four questionnaires: a translated version of the Grit Scale (Duckworth and Quinn, 2009), the Spanish versions of the Barratt Impulsiveness Scale (BISS-11; Oquendo et al., 2001) and the Five Facets Mindfulness Questionnaire (Cebolla et al., 2012), as well as the Mindful Attention Awareness Scale (Soler et al., 2012). They also underwent four experimental tasks: the Cued Task-Switching Paradigm (Chevalier et al., 2015), a Stroop-like Conflict Task (Roelofs et al., 2006), the Operation Span (Turner and Engle, 1989), and the AX-Continuous Performance Task (Braver et al., 2009). The second session included MRI and EEG recordings at rest (5 mins with eyes closed) and two experimental tasks: Stop Signal (Verbruggen and Logan, 2008) and a learning task (Anderson et al., 1994), with the latter including simultaneous EEG recordings. For the present paper, we selected the grit and impulsiveness measures as well as the EEG recordings (at rest and at task). The remaining measures are to be included in a forthcoming paper addressing related but non-overlapping research questions.

#### Grit

We translated the original Short Grit Scale into Spanish applying a back-translation method. The scale is an 8-item self-reported questionnaire that assesses two grit factors: *perseverance of effort* (i.e., “I am diligent”) and *consistency of interest* (i.e., “My interests change from year to year”). Cronbach's α of the factors of the English version is in the 0.60–0.79 range (Duckworth and Quinn, 2009). Importantly, in our sample the Cronbach's α is 0.63 for the *perseverance of effort* and 0.83 for the *consistency of interest* facets of grit.

#### BISS-11

This is a 30-item questionnaire that consists of three impulsiveness factors: cognitive impulsiveness (i.e., “I am happy-go-lucky”), motor impulsiveness (i.e., “I do things without thinking”), and non-planned impulsiveness (i.e., “I plan tasks carefully”). The Cronbach's α of the factors in this questionnaire is 0.83 (Oquendo et al., 2001).

#### Learning Task

We used an adaptation of the original selective retrieval practice task by Anderson et al. (1994) (see Valle et al., 2019) that is usually employed to investigate retrieval-induced forgetting. In this task, participants were instructed to memorize a list of words for an upcoming memory test. The task comprises 4 phases: study, practice, distraction and probe phases. In the study phase participants were instructed to memorize a list of category-exemplar pairs (54 Spanish words of nine different orthography-based categories were used; i.e., CA-Camera, CA-Casino, BA-Banana). Next, in the practice phase, they were asked to selectively retrieve half of the items of half of the categories by a given cue (i.e., CA-Cam). Then a distractor task was presented, wherein participants had to solve operational problems. In the probe phase, a recognition test was administered for all the studied items and non-studied words of different and same category. For the present work, we used the EEG signal recorded during the learning phase (5 mins). Final performance in the task was examined from the recognition index (d′) for control-baseline items (unpracticed items of unpracticed categories). Given the purpose of the present work, we focused on control-baseline items to examine overall memory performance after study rather than possible retrieval practice effects.

#### EEG Recording and Preprocessing

Participants were quietly seated with their eyes closed and the light off during the 5-min resting state EEG recording. On the other hand, to obtain the 5 mins of the task EEG measure, we chose the first 5 mins of the selective retrieval task. The selected recordings corresponded to the first 5 mins of the task during which participants were quietly seated with their eyes open, memorizing the category-word pairs. The EEG was recorded using 64 scalp electrodes that were mounted on an elastic cap using an extended 10–20 system. The continuous activity was recorded using Neuroscan Synamps2 amplifiers (El Paso, TX) and was first recorded using a midline electrode (halfway between Cz and CPz) as reference. Before data analyses, a high-pass filter at 1 Hz was applied and the 5-min recording was segmented into 2-s epochs with 0.5 s of overlap. Artifacts were manually removed by carefully inspecting the data using the Fieldtrip toolbox73 on Matlab (Oostenveld et al., 2011). Bad channels, with a high level of artifacts (always below 10% of the total for each participant), were visually detected and interpolated from neighboring electrodes.

#### Q-EEG Analyses

EEG data were analyzed using the procedures described in Prat et al. (2016). The mean log power spectrum—between 4 and 40 Hz—was calculated by first computing each epoch's power spectrum using the Fast Fourier Transform, followed by log-transforming it, and then by averaging the resulting power spectra across all epochs. To reduce spectral leakage, a Hanning window was applied to each epoch before computing the corresponding Fourier transform. The mean log power was then separately calculated across theta (4–7.5 Hz), alpha (8–12.5 Hz), beta (13–29.5 Hz), and low-gamma (30–40 Hz) frequency bands for each channel and in each participant. The frontal region of interest (ROI) was selected following Berkovich-Ohana et al. (2012): frontal (AF3, F5, F3, F1, FC3, FC1, AF4, F2, F4, F6, FC2, FC4). The theta/beta ratio was calculated for each participant by dividing the absolute theta power in the frontal cluster by the absolute beta power in the same cluster.

#### Complexity Analyses

The preprocessed EEG series were used as inputs for the Sample Entropy (SampEn) and Higuchi's Fractal Dimension (HFD) analyses. To avoid effects of change in the stability of the signal, these measures were estimated using a sliding window procedure that was 2 s in length and had a 90% overlap in each time step. The estimations were obtained from the median of the resulting complexity series for each participant, electrode, and experimental condition. SampEn represents the measure of pattern randomness in the signal. The SamplEn algorithm considers the amount of dispersion after a given time lapse between a set of closely related points in the signal. High values of SampEn are then related to time series with random structures (see seminal works of Pincus and Goldberger, 1994; Richman and Moorman, 2000). The estimation of SampEn is needed to set two free parameters (*m, p*). In our study, these were selected in accordance with the study by Richman and Moorman (2000), which recommended values of *m* = 2 and *p* = 0.10 times the SD of the series. On the other hand, the fractal dimension was estimated using the HFD algorithm (Higuchi, 1988). FD can be considered a measure of the roughness or density of the signal as depicted in a microvolt-time plot. Simple signals resembling a straight line would have a FD close to 1, while signals that tend to fill the entire space would have a FD scoring around 2. The HFD estimator of the FD takes into account the length of the signal (L) at several scales (k). The slope of the regression model for both log transformed variables (ln[k] vs ln[L]) represents the estimated FD (i.e., Ibáñez-Molina and Iglesias-Parro, 2014). Hence, the expected values for HFD are around 1.5 because 1 constitutes the minimum (values forming a straight line) and 2 a maximum (values randomly distributed as a random cloud of points) (for a review see: Kesić and Spasić, 2016). It should be noted that HFD has successfully been applied to analyses of EEG signals in both clinical and non-clinical contexts (Kesić and Spasić, 2016; Ruiz-Padial and Ibáñez-Molina, 2018). In this experiment, we selected a kmax of 55 as an optimal parameter, given that the HFD estimation approximately reached an asymptotic value for all conditions and electrodes.

## Results

Data of two participants were removed from the analyses due to artifacts during EEG recordings, while nine participants missed relevant information on the BISS questionnaire. Previous to the main analyses, we ran Shapiro-Wilk tests that confirmed that our independent variables (Grit and BISS) were normally distributed. The basic descriptive statistics are presented in Table 1. We report the results in different sections according to the goals of the study. Thus, in the first section, we report analyses testing our hypothesis about the neural correlates of grit with separate regression models in which we included the brain indexes (frontal TBR, entropy, and FD) at rest and at task as dependent variables and grit and its facets as independent variables. In the second section, we examined the relation between grit and impulsiveness in different ways. First, we report the correlation between grit and impulsiveness and tested the possible overlap between the two traits by testing hierarchical regression models. Further, we report mediation analyses to explore whether the main relationships between grit and the neural indexes were mediated by impulsiveness. Finally, we report regression models including the brain indexes (frontal TBR, entropy, and FD) at rest and at task as dependent variables and impulsiveness as the independent variable to look into the neural pattern associated with this trait and its potential similarities with grit. In the third section, we tested whether the different demographic conditions differed in grit scores. Additionally, to further explore their effects on the relationships between grit and the neural indexes, we conducted hierarchical regression analyses with the same structure than before (frontal TBR, entropy, and FD at rest and at task as the dependent variables and grit and its facets as the independent variables) but now controlling for impulsiveness, gender, age, education and work experience. For completeness, in the last section we report correlations between the main variables and performance in the learning task.

**Table 1:** Descriptive statistics for the main variables.

	**Score**	**Minimum**	**Maximum**
Grit	3.35 (0.71)	1.75	4.87
PE	14.4 (2.88)	7	20
CI	12.47 (3.7)	4	20
BISS	46.41 (13.74)	18	85
**Rest**			
Global q-EEG at rest	2.17 (0.31)	1.49	2.98
Theta q-EEG at rest	2.55 (0.32)	1.8	3.53
Beta q-EEG at rest	2.26 (0.34)	1.52	3.06
Entropy at rest	2.11 (0.04)	1.97	2.17
Fractal dimension at rest	1.69 (0.08)	1.25	1.84
**Task**			
Global q-EEG at task	2.05 (0.25)	1.41	2.69
Theta q-EEG at task	2.55 (0.23)	1.82	3.16
Beta q-EEG at task	2.09 (0.28)	1.39	2.79
Entropy at task	2.07 (0.05)	1.90	2.16
Fractal dimension at task	1.68 (0.06)	1.48	1.88
Recognition (d′) in the final stage of the selective retrieval task	1.81 (0.62)	−0.36	3

*The first column refers to means and standard deviations (SD)*.

### Electrophysiological Prints of Grit

**Figure 1:**
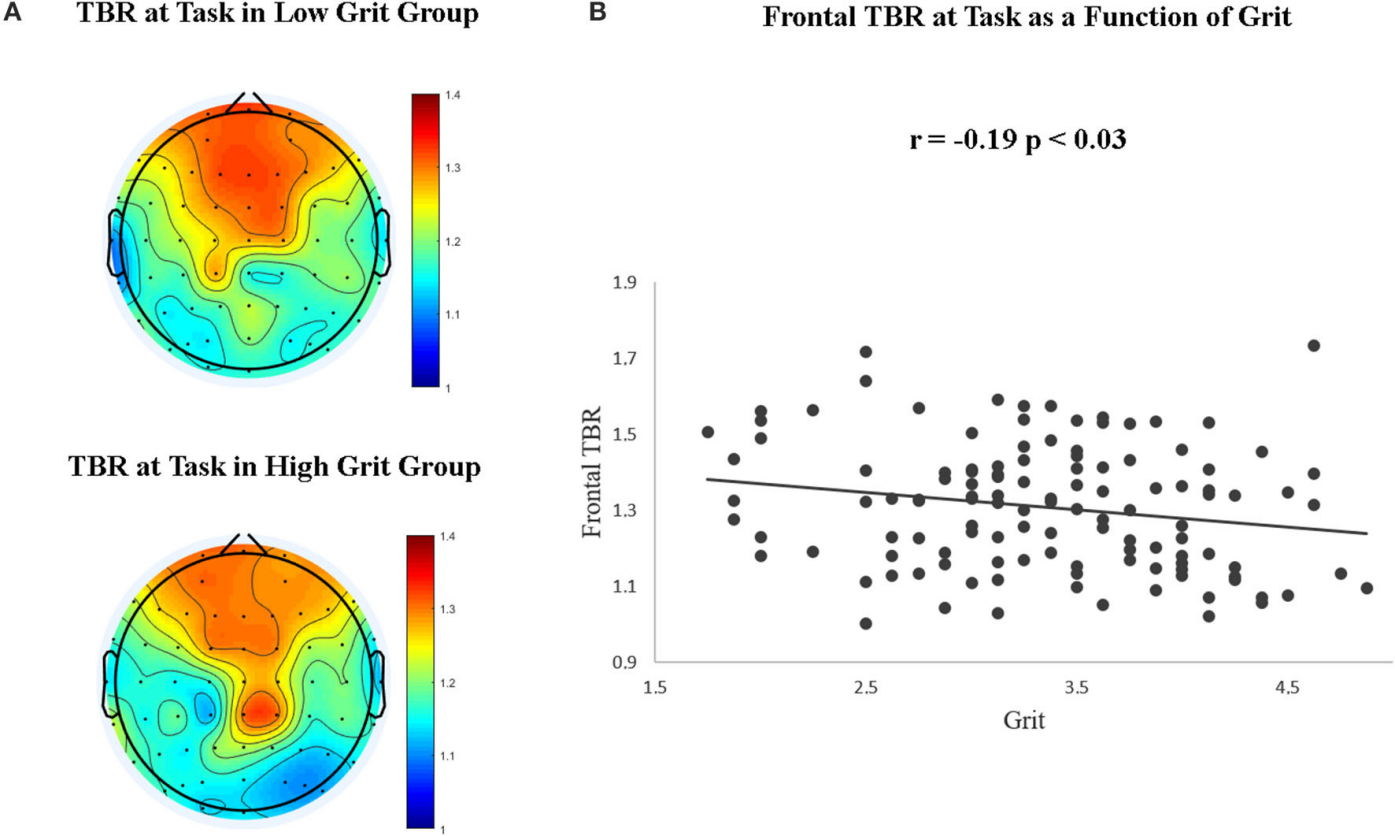
Topographical distribution of the TBR index during task performance as function of the grit group, calculated as 1 SD from the mean **(A)**, and frontal TBR as a function of grit continuous scores **(B)**.

To test our hypotheses that high grit scores were related to lower frontal TBR at rest and at task and that the *perseverance of effort* facet of grit would be related with higher complexity during task, we first ran linear regression analyses over the different neural indices (frontal TBR, entropy, and FD) at rest and at task with grit and its facets as predictors (see Table 2). The analyses showed a negative association between frontal TBR and overall grit score and *consistency of interest* (facet of grit) while performing the task. On the other hand, the analyses of complexity measures revealed a reliable (positive) association between entropy and *perseverance of effort* while performing the learning task. These associations were not evident at rest. Figure 1 plots the association between grit and lower frontal TBR at task (for a similar figure at rest see Supplementary Material 1).

**Table 2:** Linear regression analyses of grit and its two factors over neural indices (frontal TBR, entropy, and FD) during rest and during the task.

	***R*** ^**2**^	**Δ** ***F***	**B**	**SE**	**β**	***p***
**Grit**						
F TBR rest	0.00	0.05	0.11	0.48	0.02	0.82
Entropy rest	0.00	0.02	−0.78	1.72	−0.04	0.65
FD Rest	0.00	0.00	0.01	0.79	0.00	0.99
F TBR task	0.04	4.67	−0.85	0.39	−0.19	0.03
Entropy task	0.02	2.81	2.4	1.43	0.15	0.09
FD task	0.01	1.1	1.16	1.1	0.1	0.29
**PE of Grit**						
F TBR rest	0.00	0.17	0.81	1.96	0.04	0.68
Entropy rest	0.01	1.33	−8.08	7.01	−0.11	0.25
FD rest	0.01	1.03	−3.22	3.17	−0.09	0.31
F TBR task	0.00	0.58	−1.23	1.62	−0.07	0.45
Entropy task	0.04	5.11	12.96	5.73	0.21	0.026
FD task	0.01	1.03	4.55	4.48	0.09	0.31
**CI of Grit**						
F TBR rest	0.00	0.00	−0.14	−2.53	−0.01	0.96
Entropy rest	0.00	0.01	0.99	9.08	0.01	0.91
FD rest	0.01	0.69	3.41	4.09	0.08	0.41
F TBR task	0.06	7.13	−5.42	2.03	−0.24	0.009
Entropy task	0.01	0.69	6.25	7.53	0.08	0.41
FD task	0.00	0.49	4.05	5.78	0.07	0.48

### Grit and Impulsiveness

As expected, there was a negative correlation between impulsiveness and grit (*r* = −0.70, *p* < 0.001; see also **Table 7**). We examined whether the associations between brain indexes and grit were influenced by impulsiveness, by performing hierarchical regression analyses for the different neural indices (frontal TBR, entropy, and FD) at rest and at task with grit and its facets as predictors and controlling for impulsiveness (see Table 3). These analyses indicated that both the negative relation between frontal TBR at task and *consistency of interest* and the positive association between entropy at task and *perseverance of effort* were still statistically significant. In contrast, the association between frontal TBR at task and global grit score did not reach significance after controlling for impulsiveness. Additionally, we performed a mediation analysis to also examine whether the association between grit and the brain indexes was mediated by impulsiveness. These analyses indicated that impulsiveness was not a mediating factor (see Table 4). Finally, the regression model over the neural indexes with impulsiveness as the predictor failed to show any significant relationship (see Table 5).

**Table 3:** Hierarchical regression analyses of grit and its two factors over neural indices (frontal TBR, entropy, and FD) during rest and during the task controlling for impulsiveness.

	***R^2^***	***ΔF***	**B**	**SE**	**β**	***p***
**Grit**						
F TBR rest	0.00	0.01	0.00	0.02	0.01	0.9
Entropy rest	0.00	0.49	−0.00	0.00	−0.07	0.49
FD rest	0.00	0.00	0.00	0.01	0.01	0.95
F TBR task	0.03	3.39	−0.04	0.02	−0.17	0.07
Entropy task	0.01	1.28	0.01	0.01	0.11	0.26
FD task	0.01	1.57	0.01	0.01	0.12	0.21
**PE of Grit**						
F TBR rest	0.00	0.6	0.00	0.00	0.02	0.8
Entropy rest	0.01	1.57	−0.00	0.00	−0.1	0.29
FD rest	0.01	0.89	−0.00	0.00	−0.9	0.35
F TBR task	0.00	0.31	−0.00	0.00	−0.05	0.58
Entropy task	0.04	4.62	0.00	0.00	0.2	0.03
FD task	0.02	2.17	0.00	0.00	0.14	0.14
**CI of grit**						
F TBR rest	0.00	0.96	0.00	0.00	−0.00	0.96
Entropy rest	0.00	0.06	0.00	0.00	−0.02	0.81
FD rest	0.01	0.66	0.00	0.00	0.08	0.42
F TBR task	0.05	5.9	−0.01	0.00	−0.23	0.02
Entropy task	0.00	0.01	0.00	0.00	0.01	0.9
FD task	0.01	0.42	0.00	0.00	0.08	0.42

**Table 4:** Mediation analyses of consistency of interest and frontal TBR at task with impulsiveness as a mediator; and perseverance of effort and entropy at task with impulsiveness as a mediator.

	**Estimate**	**SE**	**z-value**	***p***
**CI of Grit**				
Direct effects				
Consistency of Interest → Task_F_TBR	−0.01	0.00	−2.52	0.01
Indirect effects				
Consistency of Interest → BIS → Task_F_TBR	0.00	0.0	0.82	0.41
Total effects				
Consistency of Interest → Task_F_TBR	−0.01	0.00	−2.69	0.01
**PE of Grit**				
Direct effects				
Perseverance of effort → EN_Task	0.00	0.00	2.27	0.02
Indirect effects				
Perseverance of effort → BIS → EN_Task	−6.13e −4	9.54e −4	−0.64	0.52
Total effects				
Perseverance of effort → EN_Task	0.00	0.00	2.28	0.02

**Table 5:** Linear regression analyses of impulsiveness (BISS) over neural indices (frontal TBR, entropy, and FD) during rest and during the task.

	***R^**2**^***	**Δ*F***	***B***	***SE***	**β**	***p***
**BISS**						
F TBR rest	0.00	0.00	0.00	0.00	0.03	0.75
Entropy rest	0.02	0.02	0.00	0.00	0.14	0.15
FD rest	0.02	2.35	−0.00	0.00	−0.15	0.13
F TBR task	0.01	1.06	0.00	0.00	0.1	0.30
Entropy task	0.00	0.3	0.00	0.00	−0.05	0.58
FD task	0.00	0.46	0.00	0.00	−0.07	0.50

### Grit and Demographics

Because grit has been previously related to demographic variables such as education (Duckworth et al., 2007) and work experience (Mueller et al., 2017) among others, we examined first whether such variables were linked to grit scores, and then if they could be driving the relation between grit and the neural indexes. To answer this question, we performed Pearson correlations between age and education and grit, and then *t* tests comparing men (*N* = 37) and women (*N* = 81) and people with work experience (*N* = 66) and without (*N* = 54) in their grit scores, after checking that grit was normally distributed in all groups. Results showed no association of grit with age (*r* = 0.14, *p* = 0.12), education (*r* = −0.01, *p* = 0.94), or gender [*t*_(106.66)_ = −0.9, *p* = 0.56; *M*_Males_ = 3.16; *M*_Females_ = 3.51]. However, people with work experience showed higher grit scores than people without work experience [*t*_(106.66)_ = 2.68, *p* = 0.01; *M*_Experience_ = 3.51; *M*_Non−experience_ = 3.16]. Next, we ran separate hierarchical regression analyses over the different neural indices (frontal TBR, entropy, and FD) at rest and at task with grit and its facets as predictors, now controlling for impulsiveness, gender, age and education and work experience (see Table 6). The results of these analyses showed that the negative relation between frontal TBR at task and *consistency of interest* and the positive association between entropy at task and *perseverance of effort* remained reliable.

**Table 6:** Hierarchical regression analyses of grit and its two factors over neural indices (frontal TBR, entropy, and FD) during rest and during the task controlling for impulsiveness, gender, age, education and work experience.

	***R*** ^**2**^	**Δ** ***F***	***B***	**SE**	**β**	***p***
**Grit**						
F TBR rest	0.00	0.08	0.00	0.02	0.03	0.78
Entropy rest	0.00	0.34	−0.00	0.00	−0.06	0.56
FD rest	0.00	0.01	0.00	−0.00	0.00	0.98
F TBR task	0.04	3.67	−0.03	0.02	−0.14	0.15
Entropy task	0.01	1.44	0.01	0.01	0.12	0.23
FD task	0.02	1.64	0.01	0.01	0.12	0.2
**PE of Grit**						
F TBR rest	0.00	0.42	0.00	0.00	0.06	0.52
Entropy rest	0.01	0.96	−0.00	0.00	0.09	0.33
FD rest	0.00	1.08	−0.00	0.00	−0.1	0.3
F TBR task	0.00	0.00	0.00	0.01	0.02	0.98
Entropy task	0.05	5.48	0.00	0.00	0.22	0.02
FD task	0.02	2.13	0.00	0.00	0.14	0.15
**CI of Grit**						
F TBR rest	0.00	0.00	0.00	0.00	0.00	0.97
Entropy rest	0.00	0.02	0.00	0.00	0.01	0.9
FD rest	0.00	0.58	0.00	0.00	0.07	0.45
F TBR task	0.05	6.32	−0.01	0.00	−0.23	0.01
Entropy task	0.01	0.67	0.00	0.00	0.08	0.42
FD task	0.00	0.58	0.00	0.00	0.07	0.45

### Task Performance

Finally, we performed Pearson correlation analyses between the neural indexes (TBR, entropy, FD) at task and memory performance (an index of sensitivity at recognition: d′) in the baseline condition of the selective retrieval task. These correlations did not reach statistical significance (see Table 7). We also correlated personality traits (grit and its facets, and impulsiveness) with performance, but these correlations also failed to reach statistical significance (see Table 7).

**Table 7:** Pearson correlations of the main brain variables, the Grit and BISS scores and performance in the task.

	**Grit**	**PE**	**CI**	**BISS**	**Recognition (d′)**
BISS	−0.7^***^	−0.51^***^	−0.67^***^		
Recognition (d′) in the final stage of the selective retrieval task	0.05	−0.07	0.12	−0.17	
Rest frontal TBR	0.02	0.04	−0.00	−0.04	0.01
Task frontal TBR	−0.19^*^	−0.07	−0.24^**^	0.08	−0.04
Entropy at rest	−0.04	−0.11	0.01	0.12	−0.09
Entropy at task	0.15	0.21^*^	0.08	−0.06	−0.13
Fract. Dim. at rest	−0.00	−0.09	0.08	−0.17	−0.07
Fract. Dim. at task	0.1	0.09	0.06	−0.07	−0.05

* *p < 0.05*,

** *p < 0.01*,

*** *p < 0.001. Asterisks represent statistically significant correlations after controlling for multiple comparisons with the Banjamini-Hochberg method with false discovery rate at 0.25 (Benjamini and Hochberg, 1995)*.

## Discussion

In the present study, we aimed to explore the electrophysiological prints of grit during rest and while performing a learning task. One important gap in the grit literature relates to its biological and neural substrates as only a few studies have been carried out to determine its neural mechanisms. Interestingly, despite the fact that there is little research in this area, the results converge to implicate the PFC and striatum, regions systematically associated with executive-control and motivation processes, in the expression of grit. Considering these precedents, we selected an EEG index of executive control—the frontal theta/beta ratio (TBR)—to examine its potential relationship with grit at rest and while engaged in a (learning) task. Furthermore, we selected two complexity indexes—entropy (SampEn) and fractal dimension (HDF)—to explore the possible increase in the dimensional complexity of brain activity during task performance as a function of effort employed by gritty participants. Finally, we also looked into the association between the above-mentioned EEG indexes and impulsiveness in order to determine the similarities and differences of the neural activity underlying grit and impulsiveness. Our results revealed that while there were no differences at rest as a function of grit, neural differences emerged while participants were engaged in the task. Higher overall grit and higher scores in the *consistency of interest* facet of grit were associated with lower frontal TBRs during the learning task. In addition, we observed an association between *perseverance of effort* and entropy at task, indicating that the higher the facet of grit scores are, the higher the complexity of the EEG recording is. Importantly, impulsiveness (as measured *via* the BISS) did not mediate any of the previous associations neither it was found to correlate with any of the neural indexes at rest or while performing the task. Finally, controlling for impulsiveness and demographic factors (age, gender, education and work experience) reduced the associations with overall grit scores that, however, remained statistically significant at the facet level, which highlights the relevance of these facets of grit as predictors.

The link between frontal TBR and grit during task performance is in line with results from previous studies, supporting the implication of prefrontally-mediated executive control in the grit trait (Myers et al., 2016; Wang et al., 2017, 2018). However, it is remarkable that such an association was not present at rest in our sample. TBR is an EEG index of executive control that is widely used (Arns et al., 2013; Angelidis et al., 2016; Syed Nasser et al., 2019). The ratio of theta band power (4–8 Hz) and beta band power (15–30 Hz) is thought to reflect cortical-subcortical interactions (Schutter and Van Honk, 2005; Arns et al., 2013), so that increased frontal TBR might result from a greater need for top-down control over subcortical structures (i.e., due to the triggering of inappropriate automatic responses). A large body of research suggests that mid-frontally generated theta activity is linked to activity in the anterior cingulate cortex (ACC) (i.e., Asada et al., 1999; Scheeringa et al., 2008), which is associated with more difficult situations or when reward is less than expected (Schutte et al., 2017). On the other hand, beta oscillatory activity seems to reflect active inhibitory processes involved in maintaining the current cognitive state (Engel and Fries, 2010) and is thought to be in charge of transmitting “fast-motivational signals” to downstream brain structures (Marco-Pallarés et al., 2015). Because this view aligns with the long-term-maintained motivation of gritty people, TBR could be thought of as a marker of prefrontally-mediated executive control over reward processes that are essential to grit. Such a relationship would be similar to the one reported with other subcortical processes (i.e., emotional processing, see Putman et al., 2014), although we recognize that future studies (i.e., by analyzing ERPs that are sensitive to individual differences in executive control) should more precisely determine to what extent this interpretation of the association between TBR and grit is appropriate. Interestingly, it was the *consistency of interest* facet of grit that was related to decreased frontal TBR at task, which is in line with the notion that this brain index is particularly related to the control of reward or intrinsic motivation (Putman et al., 2014). Long-term consistency of interest has been associated with more attention allocation to the current context (Aguerre et al., under review)^1^, which might allow gritty people to be more “on-task” and to avoid reward override and mind-wandering (van Son et al., 2018, 2019).

Among other results, Wang et al. (2018) found smaller gray matter volume in the left dorsolateral PFC, a region involved in self-regulation, in participants scoring high in grit. According to these authors, a reduction in gray matter would result from optimal synaptic pruning and myelination during development, which would lead to greater efficiency in corresponding psychological process (Blakemore and Robbins, 2012). Nevertheless, this finding is blind in relation to the direction of the association between grit and synaptic pruning so that grit could be either an antecedent or a consequence of greater synaptic pruning. Additionally, Wang et al. (2017) found a negative association between spontaneous brain activity in the right dorsomedial PFC and grit, which may also indicate a more efficient use of a relevant neural hub for self-regulation. Importantly, these associations were found at rest, while our study showed that differences associated with grit were particularly relevant during task performance. However, contrary to our expectations, we did not observe decreased frontal TBR in gritty participants at rest (when, in principle, there is no need for executive control). Instead, gritty participants (in their *consistency of interest* facet) exhibited lower TBR during the learning task when top-down control processes may be more crucial to keep themselves motivated. Hence, our results can also be interpreted in terms of more efficient executive functioning. When taken as a whole, our results are theoretically convergent with previous findings.

With respect to complexity measures, our results reveal that increased entropy during task performance is linked to a higher *perseverance of effort* facet of grit, but no evidence of association between the fractal dimension index and grit emerged. Entropy is a measure widely used to study self-organization and pattern formation in the complex neuronal networks of the brain (Stam, 2005). Complexity has been shown to increase during task performance (Stam et al., 1996; Bizas et al., 1999; Lamberts, 2000; Micheloyannis et al., 2002; Müller et al., 2003) as a function of the task complexity (Jie et al., 2014). Brain complexity measures have also been linked to a higher number of simultaneously activated cell assemblies, understood as representational units of thoughts and ideas (Mölle et al., 1999). Considering this evidence from previous studies, higher entropy while performing the learning task might be indicative of more effort and engagement in the task, leading to an increase in the number of activated representational units (and their corresponding cell assemblies) while memorizing a list of words. This would also fit with the idea that gritty individuals show higher general sustained attention during task performance (Kalia et al., 2018) and give support to results from previous studies using different techniques that also found *perseverance of effort* to be linked to physiological responses of effort during task (Silvia et al., 2013). In any case, we found this relationship with only one of the complexity indexes (entropy), which may be a result of the sensitivity of the measure or of these distinct measures tapping into different aspects of brain complexity (Raghavendra and Dutt, 2010; Kreuzer et al., 2014). The absence of a relationship between EEG complexity measures at rest and grit is in line with the notion that, while there probably is a stable print of grit at rest as reported by previous research (Myers et al., 2016; Nemmi et al., 2016; Wang et al., 2018), gritty people also exhibit a different and unique functional pattern that is observed only while they are engaged in a task.

On the other hand, the fact that impulsiveness did not mediate any of the associations between grit and neural indexes and that impulsiveness failed to show any relation with such indexes is also remarkable. Even when both grit and impulsiveness relate to self-regulation (Duckworth et al., 2007; Duckworth, 2011) (and they do correlate with one another, see Table 7), they exhibit a different neural pattern so reinforcing the view that they are separable constructs (Duckworth et al., 2007). On the other hand, our results concerning impulsiveness suggest that in healthy participants this trait may not involve the executive control-related neural differences that psychopathological conditions (i.e., attention-deficit hyperactivity disorder) may bring (Arns et al., 2013; Zhang et al., 2017). While higher TBR has frequently been found in impulsivity disorders, its relationship with the impulsiveness trait in healthy participants is much less clear (Lansbergen et al., 2007; Threadgill and Gable, 2018). It has been proposed that ADHD may represent the extreme end of the impulsivity continuum, characterized by increased frontal TBR, while high impulsiveness in healthy adults would involve more middle-placed positions of the continuum, characterized by average frontal TBRs (Lansbergen et al., 2007). Finally, the fact that controlling for impulsiveness and the demographic variables made global grit effects disappear but not the effects of the two facets of grit that remained significantly linked to distinct brain indexes, lends support to the different nature of the facets of grit (Credé, 2018). While this dissociation would seem to fit well with the hypotheses of the present study, *consistency of interest* correlated with a brain activity index that others have interpreted as a marker of enhanced control over reward processes (Putman et al., 2014), and *perseverance of effort* correlated with an index thought to reflect effort during the task (Stam, 2005), which is in line with the results of previous studies that used different techniques (Silvia et al., 2013), this pattern also points to the relevance of incorporating a facet level of analyses in future studies.

While consistent with the association between executive control, task engagement, and grit trait, the present findings should be taken with caution because this is one of the very first studies reporting on the electrophysiological signatures of grit. In addition, there are some considerations for future studies. First, although the TBR is an index with a relatively long history (Arns et al., 2013), its interpretation in terms of interactions between cortical and subcortical brain processes related to grit requires more research. Second, the cross-sectional design used here does not allow us to determine the direction of the association between brain indexes during task and grit. Future studies that employ longitudinal/experimental designs could help address this issue. Third, we only used self-reported measures of the traits of interest. One intriguing possibility for future studies would be to add multiple methods to assess these traits. Convergence of findings with self-reported and performance measures would be of special relevance. In this sense, future studies could add “on-task checks” and “effort checks” to experimental tasks. This could help to determine whether it is (subjective) effort that is exerted during the task and not only the general *perseverance of effort* of participants, which positively relates to entropy at task.

In sum, the present study is one of the first to unveil the electrophysiological prints of grit. Our results indicate that gritty people have a different neural signature during task, mediated by lower frontal TBR and higher entropy, which may reflect a more efficient involvement in the task. It should be noted that these results that were obtained from a large sample of young individuals with different educational and life backgrounds converge with those obtained in studies that involved children and adolescents, which goes a step further toward the generalization of findings regarding brain mechanisms of grit. While there is still a long journey ahead in order to fully understand the neural mechanisms of grit, continuing in this direction will deepen our understanding of the trait and, more importantly, potentially provide us with the empirical evidence needed to develop targeted programs and strategies to improve grit.

## Data Availability Statement

The raw data supporting the conclusions of this article will be made available by the authors, without undue reservation.

## Ethics Statement

The studies involving human participants were reviewed and approved by University of Granada. The patients/participants provided their written informed consent to participate in this study.

## Author Contributions

This work constitutes a portion of the first author's (NA) doctoral dissertation. NA, CG-A, and MB developed the concept of the study together. NA contributed to data collection, data analyses, and manuscript writing. AI-M supervised the process of data analyses and contributed to manuscript writing. MB and CG-A supervised the process of accomplishing the study and wrote, reviewed, and approved the final version of the manuscript. All authors contributed to the article and approved the submitted version.

## Funding

This research was financially supported by grants from the Spanish Ministry of Science, Innovation, and Universities and the Andalusian Government (Fondos FEDER): doctoral research grant ES-2016-078667 to NA, A-CTS-111-UGR18 and PGC2018-093786-B-I00 to MB, and PSI2015-65502-C2-2-P to CG-A.

## Conflict of Interest

The authors declare that the research was conducted in the absence of any commercial or financial relationships that could be construed as a potential conflict of interest.

## Publisher's Note

All claims expressed in this article are solely those of the authors and do not necessarily represent those of their affiliated organizations, or those of the publisher, the editors and the reviewers. Any product that may be evaluated in this article, or claim that may be made by its manufacturer, is not guaranteed or endorsed by the publisher.

## Footnotes

^1^Aguerre, N. V., Gómez-Ariza, C. J. and Bajo, M. T. (under review). The relative role of executive control and personality traits in grit.
