# Supplementary material for: Electrophysiological Prints of Grit
Source: Front Psychol. 2021 Oct 14;12:730172. doi: 10.3389/fpsyg.2021.730172 (PMC8551368; doi:10.3389/fpsyg.2021.730172)
Supplement: Supplementary file 1 [file Image_1.pdf]

Supplementary Material 1

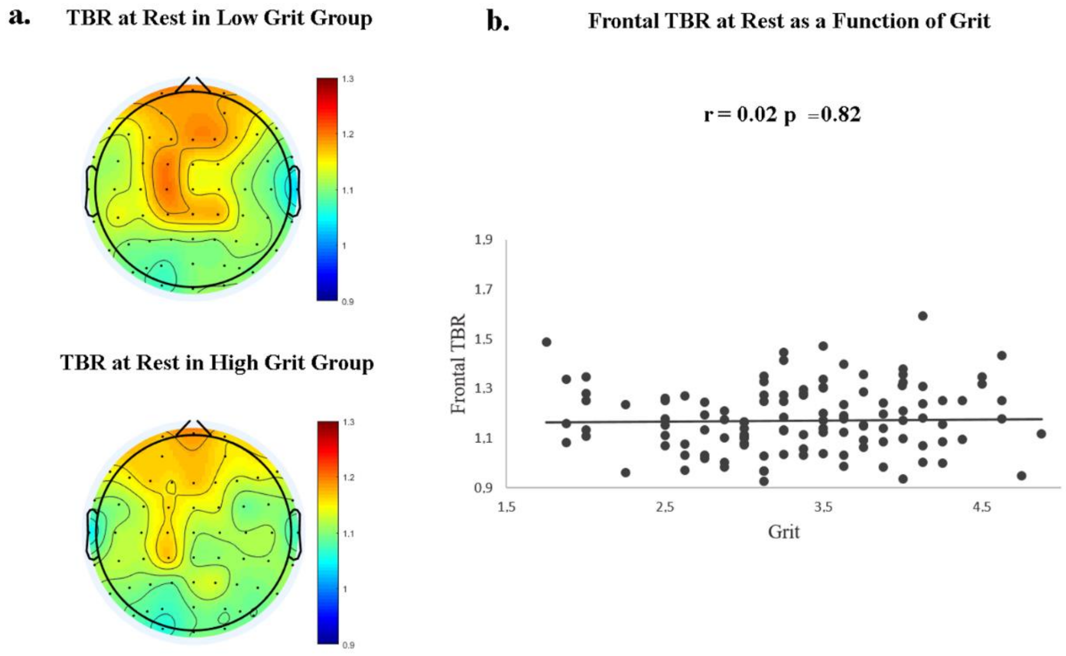

Fig. 1. Topographic representation of the TBR index during rest performance as function of the grit group, calculated as 1 SD from the mean (a), and frontal TBR as a function of grit continuous scores (b).
